# The contribution of school meals to energy and nutrient intake of Swedish children in relation to dietary guidelines

**DOI:** 10.3402/fnr.v59.27563

**Published:** 2015-10-30

**Authors:** Christine Persson Osowski, Anna Karin Lindroos, Heléne Enghardt Barbieri, Wulf Becker

**Affiliations:** 1 Department of Food, Nutrition and Dietetics, Uppsala University, Uppsala, Sweden; 2 National Food Agency, Uppsala, Sweden

**Keywords:** school meals, school lunches, children, nutrient intake, energy intake

## Abstract

**Background:**

In Sweden, school meals are served free of charge and Swedish law states that school meals must be nutritious. Nevertheless, data on children's energy and nutrient intake from school meals are scarce.

**Objective:**

The aim was to describe the contribution of school meals to Swedish children's nutrient and energy intake during weekdays and compare this to the reference values based on the Nordic Nutrition Recommendations (NNR), which have been adopted as the official Swedish recommendations.

**Design:**

A cross-sectional food consumption survey was performed on 1,840 Swedish children attending Grade 2 (mean age 8.6) and Grade 5 (mean age 11.7). The children's nutrient and energy intake was compared to the reference values based on the NNR.

**Results:**

The mean intake from school meals of energy, carbohydrates, dietary fiber, polyunsaturated fatty acids (PUFA), and vitamins D and E did not reach the reference values and the intake of saturated fatty acids (SFA) and sodium exceeded the reference values in both age groups (significant differences, all *p*≤0.001). Additionally, the pupils in Grade 5 did not reach the reference values for folate, potassium, calcium, magnesium, iron, selenium, and zinc (significant differences, all *p*≤0.001). Standardized for energy, dietary fiber, PUFA, and vitamins D and E did not reach the reference values, whereas the reference values for SFA and sodium were exceeded in both age groups (significant differences, all *p*≤0.001).

**Conclusions:**

The study pointed to some central nutrients in need of improvement as regards school meals in Sweden, namely the quality of fat, dietary fiber, sodium, vitamin D, and iron. Some of these results may be attributed to the children not reporting eating the recommended number of calories, the children omitting some components of the meal, or underreporting, as a consequence of which the reference values for several nutrients were not met.

School food is a topic of discussion worldwide. In Europe, all European Union member countries as well as Norway and Switzerland have either voluntary guidelines or mandatory school food policies regarding what may be served in the school setting. The aims of these school food policies are in a majority of cases to improve child nutrition, to teach healthy diet and lifestyle habits, and to reduce or prevent childhood obesity (1). In Sweden, school meals are served free of charge to all children in compulsory school. School meals in Sweden often consist of one or more cooked dishes to choose from (usually consisting of meat, fish, poultry, or a vegetarian alternative, served with rice, potatoes, or pasta), vegetables, milk or water to drink, and crisp bread with spread. ‘Competitive foods’ sold in cafeterias or vending machines are rare and there is no tradition of children bringing their own lunch to school.

The health perspective has been evident ever since the introduction of school meals in Sweden in 1946. Back then, the goal was to address and deal with the issue of malnutrition among children (2), offering a free meal to all children, whereas today, the problem is rather energy imbalance. *Riksmaten barn*, the 2003 Swedish national food consumption survey conducted by the National Food Agency (NFA), showed that about every fifth child is overweight or obese and that 25% of the participating children's energy intake came from energy-dense foods of poor nutritional quality (3). In 2011, a law was enacted in Sweden stating that school meals should be nutritious (4). This law requires that the nutritional content of school meals be ensured and documented (5). The NFA publishes guidelines that aim to support schools in their work with school meals (6). The guidelines elaborate on the prevalence of unhealthy eating habits among children found in *Riksmaten barn*, noting that part of the problem is that many pupils do not eat enough of the school lunch. Thus, not only is it important to make school lunches sufficiently nutrient dense, but also to make the food and meal situation appealing enough for the children to eat a complete school lunch, in order to limit intake of food items of poor nutritional quality during the day.

The guidelines recommend calculation of the nutritional content of all meals served over a time period of 4 weeks. Over this 4-week period, an average school lunch should provide for about 30% of the recommended energy intake and dietary reference values (DRVs) for vitamins and minerals stated in the Nordic Nutrition Recommendations (NNR) (7), which are also the official Swedish nutrition recommendations. For the sake of simplicity, the NFA guidelines have chosen to focus on the nutrients that are considered of special importance for children, namely energy, total fat, saturated fatty acids (SFA), polyunsaturated fatty acids (PUFA), carbohydrates, protein, dietary fiber, vitamin C, vitamin D, folate, iron, and sodium.

Internationally, there have been studies published in the past few years regarding the nutritional quality of school meals and children's energy and nutrient intake from school meals, for example from Finland (8), the United States (9), and Great Britain (10, 11). These are countries that, like Sweden, have some form of requirements regarding the nutritional quality of school meals (5). Although the Swedish context with its free school meals ought to be of special interest to study, similar up-to-date studies from Sweden are lacking. Older studies from Sweden have shown that pupils often eat less than the recommended 30% of the daily energy intake for lunch. Nonetheless, the contribution of protein, fat, and carbohydrates was fairly balanced in relation to the Swedish nutrition recommendations (12). A recent survey conducted in 2012–2013 based on a web-based system, allowing schools to evaluate their school food provision without nutritional calculations, studied whether schools are likely to reach the recommendations regarding dietary fiber, iron, vitamin D, and quality of fat, that is, the proportion of energy derived from SFA and PUFA. The results indicated that a majority of the participating schools are likely to reach the recommendations for dietary fiber and iron (91 and 83%, respectively), but that only a minority are likely to reach the recommendations for vitamin D and quality of fat (36 and 21%, respectively). Altogether, 12% of the participating schools fulfilled all four nutritional criteria (13). Other than this, data on Swedish school meal nutritional quality are scarce. The present study is based on secondary analyses of data from *Riksmaten barn*, the national food consumption survey of children previously mentioned (3, 14). The aim was to describe the contribution of school meals to Swedish children's nutrient and energy intake during weekdays and compare this to the reference values based on the NNR.

## Methods

In the spring and autumn of 2003, the national food consumption survey *Riksmaten barn* was conducted by the NFA using data from children aged 4 years as well as schoolchildren attending Grade 2 (usually aged 8–9 years) and Grade 5 (usually aged 11–12 years) in Sweden. A more detailed description of the methods used and general results of this study have been presented elsewhere (3, 14). The present article is based on secondary analyses of the data covering the schoolchildren, aiming to describe the contribution of school meals to the children's nutrient and energy intake on weekdays.

### Selection of participants

According to Swedish law, the study was not in need of ethical approval. However, headmasters and subsequently each child's parents gave written consent for participation in the study. The children were selected from a nationally representative cluster sample of Swedish municipalities. From the nationally representative cluster of municipalities, schools and classes of second graders and fifth graders from these schools were randomly selected. In total, 2,499 school children were asked to participate (1,209 children in Grade 2 and 1,290 in Grade 5). Of the 2,232 children who agreed to participate, 1,945 children completed the survey. Of these, 40 were excluded due to incomplete food diaries. All in all, 1,905 children, that is, 76% of the original sample, were included in the original study and thus eligible for these secondary analyses. Children who declined were more likely to be boys, have a foreign background, live in a single-parent household, and have siblings than those who participated.

### Data collection

The method used in the data collection was a food diary. All food items and beverages consumed by the children were recorded in an open, estimated food diary for four consecutive days with all days of the week evenly represented in the sample. The food diary and study design were pilot tested before the actual data collection took place. The participants were given both oral and written information about the study. The food diary was completed by the children themselves or with the help of an adult. All foods and beverages consumed were recorded, together with the time and place for each eating occasion. A picture booklet was provided in order to help the participants estimate portion sizes and amounts of food. The amounts consumed were also estimated using household measures such as glasses, deciliters, and so forth or were recorded in grams. The children also completed a questionnaire on background information and question items covered areas such as meal patterns and food choice (14). For the purpose of the present study, mainly the background data were used. Those who did not return their food diary or questionnaire received one reminder. Each school class with a participation rate of at least 80% was given a reward of SEK 2000 (equivalent to about EUR 217 or USD 278). In the present study, the background variables of age, sex, weight, height, regional residence, ethnicity, and parents’ education as well as reported school meal consumption habits were described. Furthermore, reported energy and nutrient intakes from school meals and the whole day when school meals were eaten were used for the school meal analyses.

### Analyses

All food items and beverages recorded were coded using the food composition database of the NFA, version 04.1.1 (15). All estimated amounts of food eaten were converted to grams. The analyses of nutrient and energy intakes were performed using the computer software MATs (16). These tasks were all performed by staff at the NFA.

SPSS version 20.0 was used for the statistical analyses (IBM Corporation, Armonk, NY, USA); *p*<0.05 was considered significant. Multiple tests were performed; thus the total significance level was larger than the 5% used in a single test, and the significance of the different test results must be interpreted with care.

Only children who had eaten at least one school meal on any of the recorded days were included. A Pearson chi-square test was used to assess whether there were any differences regarding background variables between children included (*n*=1,840) and excluded (*n*=65) from the study. When data were missing, cases were excluded analysis by analysis. Since all assumptions of the chi-square test were fulfilled, exact tests were not required. Thus, the asymptotic method, which is the standard method in SPSS, was used.

For the purpose of this study, only meals that had been recorded as lunch eaten at school were included. As previously mentioned, school lunches are served for free in Sweden, and therefore there is no tradition of children bringing lunch to school. Lunches consumed elsewhere and lunches that were consumed on weekends were excluded. However, alternatives such as meals consumed at fast food restaurants or that had been bought from grocery stores were few and a majority of the children in the present study reported that they ate the school lunch every day. The study participants recorded consumption of one to four school meals. Therefore the mean intake was calculated for each participant and used in the analyses. All food items and beverages consumed were included in the calculations, that is, the main course and additional components of the school lunch such as milk, vegetables, and bread with spread. The children's energy and nutrient intakes from the school meals are presented both as actual mean daily intake and standardized for energy, that is, nutrient density (*per* MJ). The nutrient density of the school meals consumed was calculated as nutrient values for each subject divided by energy content for that subject (17) for dietary fiber and all vitamins and minerals. Energy percentage (E%) is presented for all macronutrients. Based on the mean intakes from school meals and in total during the day, the contribution of school meals to the children's total energy and nutrient intakes during days when school meals were eaten were calculated and are presented as a percentage of daily intake.

It is customary to use average requirement and lower intake level values to assess results from dietary surveys, but these values are only available for adults in Sweden (7). Therefore, the children's mean energy and nutrient intakes were compared to the current Swedish guidelines for school meals (6). These are based on 30% of the DRVs in the NNR (7). However, it must be stressed that these values are mainly intended to be used when planning meals and not to assess whether individuals reach the requirements. Moreover, the data were collected before the current guidelines were implemented. Two-sided, one-sample *t*-tests were used to test whether the population means differ from the reference values stated in the NNR. The variables were not normally distributed and the sample contained outliers. However, considering the large sample size, lack of normality should not have constituted a problem (18, 19) and due to the fact that the means and 5% trimmed means were similar, the outliers should not have affected the results in a major way (18). Both actual intake and intake standardized for energy were tested. The tests were performed separately for the two age groups included, since the NNR DRVs differ for these two groups. Means and 95% confidence intervals of the mean intake are presented instead of confidence intervals of the difference, and thus insignificant results do not include zero in the confidence intervals.

The reference values stated in the guidelines for school meals, which are equivalent to 30% of the NNR DRVs, were used as test values in the two-sided, one-sample *t*-tests (6) (see Tables 2–4 for information on the test values used). As previously mentioned, the guidelines only state reference values for some nutrients. Therefore, for all other nutrients included in the present study, 30% of the DRVs in the NNR were calculated. Energy and energy-providing nutrients are stated as ranges in the guidelines and NNR. Here the lowest value in that reference was chosen in order to determine whether school lunches fulfill the minimum requirement for macronutrients, except for total fat, for which a limit of the highest reference value was used. As for energy, 30% of the children's recommendation for energy was chosen, as this is the standard used in the guidelines for school meals. Thus, the hypotheses tested were that the mean intake of energy was not below the reference value equivalent to 30% of the recommended energy intake, that total fat did not exceed the upper intake range, and for all other macronutrients that the mean intake was not below the lower reference value in the interval. For the other nutrients with a set reference value, the hypotheses tested were that the mean intake of SFA, sucrose, and sodium did not exceed the reference value and for all the remaining nutrients that the mean intake was not below the reference value. The NNR include a recommendation to limit the intake of added sugars (e.g. mono- and disaccharides and various isolated sugar preparations) to less than 10 E%. In the present study, intake of sucrose was used as a proxy for added sugars, since the food composition database lacked data on added sugars. Part of the sucrose intake comes from natural sources, and therefore the results should be used with some caution. The reference values differ between boys and girls aged 10–13 years for some nutrients, and in these cases the reference value for the group with the highest need was chosen, which is the same principle used in the guidelines for school meals (5). When the children's intake standardized for energy was tested, the reference values in the NNR were adjusted to the energy reference values for school meals (i.e. 2.1 MJ and 2.7 MJ for the two included age groups, respectively).

## Results

### Participants

Of the 1,905 children who were included in the original study, 65 (3%) of these participants did not eat a school meal on any of the recorded days. They were therefore excluded and thus 1,840 children were included in these secondary analyses of the nutrition data. The mean age (standard deviation) of the participants in the present study was 8.6 (0.4) years in Grade 2 and 11.7 (0.4) years in Grade 5. Characteristics of the excluded and included children are presented in Table 1. The total number of school meals recorded for these individuals was 4,779. Overall, 20% (*n*=362) of the children recorded four school lunches, 30% (*n*=549) three school lunches, 41.0% (*n*=755) two school lunches, and 9% (*n*=174) one school lunch.

**Table 1:** Characteristics of and differences between the subjects who were excluded from the study, that is, for whom school meal data were lacking, and included in the study, that is, who completed the survey and had eaten at least one school meal on any of the registered days

	Excluded		Included				
					
Variable	*n*=65	%	*n*=1,840	%	χ^2^	df	*P* a
Grade							
2	31	48	858	47			
5	34	52	982	53	0.028	1	0.866
Gender							
Male	27	42	934	51			
Female	38	58	906	49	2.136	1	0.144
Area of residence							
Big city	26	40	608	33			
Medium-sized city	13	20	415	23			
Rural area	26	40	817	44	1.370	2	0.504
Ethnicity^b^							
Child and parent born in Sweden	38	62	1,408	79			
Parent or child born outside Sweden	23	38	378	21	9.494	1	0.002*
BMI^c^							
BMI<25	52	95	1,370	82			
BMI≥25	3	5	293	18	5.524	1	0.019*
Parents’ education^d^							
High school or lower	37	59	961	54			
College/university	26	41	835	46	0.668	1	0.414
School meal consumption^e^							
Always has school lunch	49	78	1,590	88			
Has school lunch fewer than five times/week	14	22	211	12	6.331	1	0.012*
Omission of main meal^f^							
Always has the main meal	19	48	866	68			
Omits the main meal at least once/week	21	53	406	32	7.483	1	0.006*

* Indicates significant differences.

a The chi-square test was used to determine whether there were any differences regarding the background variables between included and excluded children;

b 58 (3%) observations missing;

c age-adjusted body mass index (187 (10%) observations missing);

d classified by the parent with the highest education level in the household (46 (2%) observations missing);

e 41 (2%) observations missing;

f how many times/week the pupils omit the main meal when having school lunch, that is, only have bread, salad, a beverage, and/or sour milk for lunch. A total of 593 (31%) observations were missing. Because the numbers have been rounded off, the percentages do not always add up to 100%.

There were some differences in background data between the children who were included and excluded from the study (Table 1). In the group of 65 children for whom school meal data were lacking on any of the recorded days, there were significantly more children of foreign background (38% as compared to 21%, *p*=0.002), significantly fewer children with a BMI≥25 (5% as compared to 18%, *p*=0.019), significantly more children who sometimes skip school lunch (22% as compared to 12%, *p*=0.012), and significantly more children who omit the main meal at least once/week when having school lunch, that is, who only have bread, salad, a beverage, and/or sour milk for lunch (53% as compared to 32%, *p*=0.006), than among the children included in the study.

### Contribution to total energy and nutrient intake

**Fig. 1:**
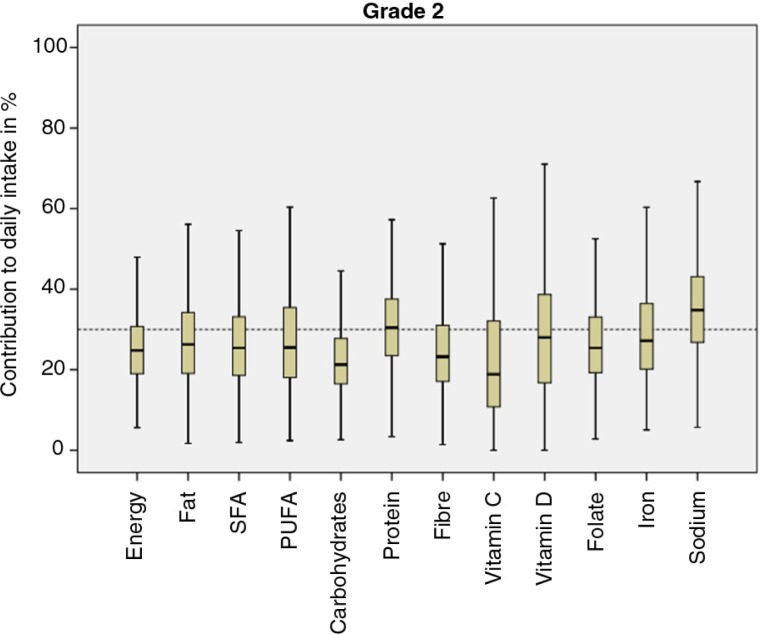
Boxplots describing the contribution of energy and nutrients from school lunches to daily intakes in percent for children in Grade 2 for the nutrients included in the Swedish guidelines for school meals. The dotted reference line represents 30% of the children's daily intake. SFA, saturated fatty acids; PUFA, polyunsaturated fatty acids.

**Fig. 2:**
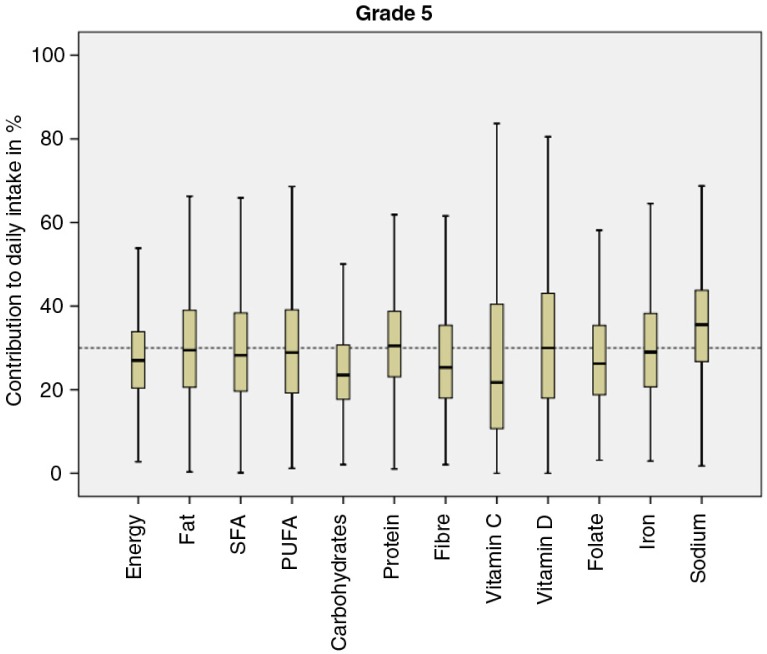
Boxplots and means describing the contribution of energy and nutrients from school lunches to daily intakes in percent for children in Grade 5 for the nutrients included in the Swedish guidelines for school meals. The dotted reference line represents 30% of the children's daily intake. SFA, saturated fatty acids; PUFA, polyunsaturated fatty acids.

The children in Grade 2 consumed a mean total of 7,554 kJ and the children in Grade 5 a total of 7,140 kJ on the recorded weekdays. These figures can be compared to the estimated average reference values for children in the NNR (based on average weight and physical activity), which are 6,780 kJ/7,270 kJ for girls and boys, respectively, in Grade 2 and 8,360 kJ/8,900 kJ for girls and boys, respectively, in Grade 5. Results regarding contribution to total energy and nutrient intake for the nutrients included in the Swedish guidelines for school meals for children in Grade 2 and Grade 5 are displayed in Figs. 1 and 2. At the group level, that is, with both age groups included, the children received about 27% of their daily energy intake from school meals. Where nutrient intakes were concerned, the contribution to the daily intake was either equal to or higher relative to the energy intake, that is, above 27% (total fat 29%, SFA 28%, PUFA 29%, protein 31%, vitamin D 31%, folate 27%, iron 30%, and sodium 36%), with the exceptions being carbohydrate (24%), vitamin C (26%), and dietary fiber (26%). As for sucrose, school meals contributed to about 11% of the children's daily intake (not included in the guidelines). However, these results apply at the group level. The boxplots displayed in Figs. 1 and 2 indicate that they can vary to a great extent at the individual level and to some extent between the two age groups, with the children in Grade 5 showing a tendency to consume a larger proportion of their daily intake of energy and nutrients from school meals than the children in Grade 2.

### Energy and nutrient intakes as compared to the NNR

The mean intake of energy and nutrients from the school lunch compared to the reference values based on the NNR are presented in Tables 2–4. The mean intake of energy, carbohydrates, dietary fiber, PUFA, vitamin D, and vitamin E did not reach the reference values and the intake of SFA and sodium exceeded the reference values in both age groups (significant differences, all *p*≤0.001). In addition, the pupils in Grade 5 did not reach the reference values for folate, potassium, calcium, magnesium, iron, selenium, and zinc (significant differences, all *p*≤0.001). The mean content of nutrients standardized for energy compared to the NNR-based energy-standardized reference values (2.1 MJ for children in Grade 2 and 2.7 MJ for children in Grade 5) are presented in Tables 2–4. Standardized for energy, dietary fiber, PUFA, and vitamins D and E did not reach the reference values, whereas the reference values for SFA and sodium were exceeded in both age groups (significant differences, all *p*≤0.001). If we look at the confidence intervals, children eating school meals are likely to fall short of the NNR-based reference values by approximately 1 g/MJ for dietary fiber, 1 E% for PUFA, 0.5 µg/MJ for vitamin D, and 0.1 mg/MJ or less for vitamin E and to exceed the reference values for SFA by approximately 5 E% and in the range of 247–335 mg/MJ for sodium. The presented intake of sucrose includes both added sugars and natural sources of sucrose. In spite of this, the intake of sucrose was below the NNR reference value of <10% added sugars.

**Table 2:** Actual mean intake and intake standardized for energy (E% or *per* MJ) for energy and macronutrients from school meals among pupils in Grade 2 and Grade 5 compared to the Swedish reference values

		Pupils in Grade 2 (*n=*858)				Pupils in Grade 5 (*n=*982)			
			
Energy and macronutrients		Reference values^a^	Mean	95% CI (lower, upper)^b^	*P* c	Reference values^a^	Mean	95% CI (lower, upper)^b^	*P* c
Energy	kJ	2,100	1,889	1,838, 1,941	<0.001*	2,700	1,944	1,891, 1,997	<0.001*
Protein	g	12	22.1	21.4, 22.8	<0.001	16	20.7	20.1, 21.3	<0.001
	E%	10–20	20.1	19.7, 20.4		10–20	18.3	18.0, 18.6	
Carbohydrates	g	55	52.1	50.6, 53.7	<0.001*	71	53.3	51.7, 54.9	<0.001*
	E%	45–60	47.3	46.7, 47.9		45–60	47.7	46.9, 48.4	
Sucrose^d^	g	<12	4.2	3.8, 4.6	<0.001	<16	4.9	4.4, 5.4	<0.001
	E%	<10	3.8	3.4, 4.1		<10	4.1	3.7, 4.4	
Dietary fiber	g	6	3.5	3.4, 3.6	<0.001*	8	3.4	3.3, 3.5	<0.001*
	g/MJ	3	1.9	1.9, 2.0	<0.001*	3	1.9	1.9, 2.0	<0.001*
Total fat	g	≤22	17.4	16.8, 17.9	<0.001	≤29	18.8	18.1, 19.4	<0.001
	E%	25–40	33.6	33.0, 34.1		25–40	34.5	33.9, 35.2	
SFA	g	≤6	7.7	7.4, 7.9	<0.001*	≤7	8.1	7.8, 8.4	<0.001*
	E%	≤10	14.9	14.6, 15.1		≤10	14.9	14.6, 15.2	
MUFA	g	6	6.4	6.2, 6.6	0.001	7	7.2	6.9, 7.4	0.258
	E%	10–20	12.3	12.0, 12.5		10–20	13.1	12.8, 13.4	
PUFA	g	3	2.1	2.0, 2.2	<0.001*	4	2.2	2.1, 2.3	<0.001*
	E%	5–10	4.0	3.9, 4.2		5–10	4.2	4.0, 4.3	

* Indicates significant differences that deviate from the recommendations. SFA, saturated fatty acids; MUFA, monounsaturated fatty acids; PUFA, polyunsaturated fatty acids; CI, confidence interval.

a The reference values, which are different for the two age groups, are based on the Swedish guidelines for school meals (6) when applicable, or 30% of the NNR (7) reference values;

b 95% confidence intervals of the mean intake are presented. Please note that since confidence intervals for the mean intake are presented instead of confidence intervals for the mean difference, insignificant results will not include zero;

c two-sided, one-sample *t*-tests were used to determine whether the population mean differs from the reference values. Since two-sided tests were used, deviations of the estimated parameter are detected in either direction. Therefore only significant differences that deviate from the recommendations are marked with an asterisk (*);

d the reference value refers to added sugars, but the presented intakes include both added sugars and natural sources of sucrose.

**Table 3:** Actual mean intake and intake standardized for energy (*per* MJ) for vitamins from school meals among pupils in Grade 2 and Grade 5 compared to the Swedish reference values

		Pupils in Grade 2 (*n=*858)				Pupils in Grade 5 *(n=*982)			
			
Vitamins		Reference values^a^	Mean	95% CI (lower, upper)^b^	*P* c	Reference values^a^	Mean	95% CI (lower, upper)^b^	*P* c
Vitamin A^d^	µg	120	328	311, 345	<0.001	180	245	232, 259	<0.001
	µg/MJ	57	188	178, 198	<0.001	67	137	128, 147	<0.001
Vitamin D	µg	3.0	1.5	1.4, 1.6	<0.001*	3.0	1.5	1.4, 1.5	<0.001*
	µg/MJ	1.4	0.8	0.8, 0.8	<0.001*	1.1	0.8	0.7, 0.8	<0.001*
Vitamin E^e^	α-TE	1.8	1.6	1.5, 1.6	<0.001*	2.4	1.5	1.5, 1.6	<0.001*
	α-TE/MJ	0.9	0.8	0.8, 0.9	<0.001*	0.9	0.8	0.8, 0.8	<0.001*
Vitamin C	mg	12	17	16, 18	<0.001	15	16	15, 16	0.262
	mg/MJ	6	9	9, 10	<0.001	6	9	8, 10	<0.001
Thiamin	mg	0.27	0.42	0.40, 0.44	<0.001	0.33	0.38	0.37, 0.40	<0.001
	mg/MJ	0.13	0.23	0.22, 0.23	<0.001	0.12	0.21	0.20, 0.22	<0.001
Riboflavin	mg	0.33	0.43	0.42, 0.45	<0.001	0.39	0.41	0.40, 0.43	0.003
	mg/MJ	0.16	0.23	0.23, 0.24	<0.001	0.14	0.21	0.21, 0.22	<0.001
Folate	µg	39	52	51, 54	<0.001	60	49	47, 51	<0.001*
	µg/MJ	19	28	28, 29	<0.001	22	27	26, 27	<0.001
Vitamin B_12_	µg	0.4	1.5	1.4, 1.6	<0.001	0.6	1.4	1.4, 1.5	<0.001
	µg/MJ	0.2	0.8	0.8, 0.8	<0.001	0.2	0.7	0.7, 0.8	<0.001
Pyridoxine	mg	0.3	0.5	0.5, 0.6	<0.001	0.4	0.5	0.5, 0.5	<0.001
	mg/MJ	0.1	0.3	0.3, 0.3	<0.001	0.1	0.3	0.3, 0.3	<0.001

* Indicates significant differences that deviate from the recommendations.

a The reference values, which are different for the two age groups, are based on the Swedish guidelines for school meals (6) when applicable, or 30% of the NNR (7) reference values;

b 95% confidence interval (CI) of the mean intake is presented. Please note that since confidence intervals for the mean intake are presented instead of confidence intervals for the mean difference, insignificant results will not include zero;

c two-sided, one-sample *t*-tests were used to determine whether the population mean differs from the reference values. Since two-sided tests were used, deviations of the estimated parameter are detected in either direction. Therefore, only significant differences that deviate from the recommendations are marked with an asterisk (*);

d retinol equivalents;

e α-tocopherol equivalents.

**Table 4:** Actual mean intake and intake standardized for energy (*per* MJ) for minerals from school meals among pupils in Grade 2 and Grade 5 compared to the Swedish reference values

		Pupils in Grade 2 (*n=*858)				Pupils in Grade 5 (*n=*982)			
			
Minerals		Reference values^a^	Mean	95% CI (lower, upper)^b^	*P* c	Reference values^a^	Mean	95% CI (lower, upper)^b^	*P* c
Potassium	mg	600	898	871, 924	<0.001	990	861	836, 887	<0.001*
	mg/MJ	286	490	480, 500	<0.001	367	462	452, 473	<0.001
Phosphorus	mg	162	368	357, 379	<0.001	210	354	344, 365	<0.001
	mg/MJ	77	198	194, 201	<0.001	78	185	182, 189	<0.001
Calcium	mg	210	254	244, 264	<0.001	270	241	231, 252	<0.001*
	mg/MJ	100	137	133, 142	<0.001	100	124	120, 129	<0.001
Magnesium	mg	60	70	68, 72	<0.001	84	68	66, 70	<0.001*
	mg/MJ	29	38	37, 38	<0.001	31	36	36, 37	<0.001
Iron	mg	2.7	2.6	2.5, 2.7	0.196	3.3	2.4	2.3, 2.5	<0.001*
	mg/MJ	1.3	1.4	1.3, 1.5	0.001	1.2	1.3	1.2, 1.3	<0.001
Selenium	µg	9	11	10, 11	<0.001	12	10	10, 10	<0.001*
	µg/MJ	4	6	6, 6	<0.001	4	5	5, 5	<0.001
Zinc	mg	2.1	3.2	3.1, 3.3	<0.001	3.3	3.0	2.9, 3.1	<0.001*
	mg/MJ	1.0	1.7	1.7, 1.7	<0.001	1.2	1.6	1.5, 1.6	<0.001
Sodium	mg	≤480	1,026	992, 1,061	<0.001*	≤720	994	963, 1,025	<0.001*
		(≤1.2 g salt)				(≤1.8 g salt)			
	mg/MJ	≤229	552	540, 564	<0.001*	≤267	525	514, 536	<0.001*

* Indicates significant differences that deviate from the recommendations.

a The reference values, which are different for the two age groups, are based on the Swedish guidelines for school meals (6) when applicable, or 30% of the NNR (7) reference values;

b 95% confidence interval (CI) of the mean intake is presented. Please note that since confidence intervals for the mean intake are presented instead of confidence intervals for the mean difference, insignificant results will not include zero;

c two-sided, one-sample *t*-tests were used to determine whether the population mean differs from the reference values. Since two-sided tests were used, deviations of the estimated parameter are detected in either direction. Therefore, only significant differences that deviate from the recommendations are marked with an asterisk (*).

## Discussion

In line with previous studies in Sweden (12), the intake from school meals did not reach the reference values included in the guidelines for carbohydrates, dietary fiber, PUFA, and vitamin D, but exceeded the reference values for SFA and sodium. For the older age group, school meal reference values for folate and iron were also not met. This was also the case for some nutrients not covered in the guidelines, for instance vitamin E in both age groups. However, many of these findings were not consistent when the nutrient intakes were standardized for energy. School meals provided on average 27% of the daily energy intake, which is somewhat less than the 30% stated in the guidelines. School meals would have provided sufficient amounts of nutrients, except for dietary fiber, PUFA, and vitamins D and E, had the children only consumed the amount of energy recommended in the guidelines. Moreover, SFA and sodium remain problem areas. The findings support the choice of key nutrients included in the school meal guidelines.

The results were largely comparable to the *Riksmaten barn* national food consumption survey as a whole (3) and the previously mentioned survey on nutritional quality of school meals (13), which pointed out certain nutrients in need of improvement. These would primarily be the quality of fat, dietary fiber, sodium, vitamin D, and iron. The present study also showed low intakes of vitamin E. Nevertheless, vitamin E–deficiency is rare (7) and, when standardized for energy, the confidence intervals indicate that school meals’ contribution to vitamin E is close to the DRVs. Intake of vitamin E generally is correlated with PUFA intake, as most foods that contain a lot of PUFA are also rich in vitamin E (7). Thus, a higher content of PUFA in school meals, thereby reaching the guideline reference value, would probably be accompanied by higher vitamin E content.

The quality of fat was not satisfactory; the proportion of SFA was higher and the proportion of PUFA was lower than the reference values. The Swedish guidelines for school lunches (6) include concrete advice on how the quality of fat in school lunches may be attained. If these guidelines had been followed, the results would most likely look different.

The NNR acknowledges that a sufficient amount of dietary fiber is also important for children (7). Nevertheless, the DRV of 3g/MJ was not reached in the present study. School meals in Sweden are usually served with rye crispbread and vegetables, which are important sources of dietary fiber. However, since children typically serve themselves at the school lunch, their dietary fiber intake is most likely dependent on whether or not they choose to eat these complementary food items.

The children received on average 36% of their daily sodium intake from the school lunch, and the intake well exceeded the guidelines. Nevertheless, there is uncertainty regarding the sodium content of the food, because the amount of sodium added during cooking may vary. Moreover, the guidelines acknowledge that the reference value for sodium may currently be difficult to achieve in practice and it is therefore stated to be a long-term goal (5, 6). In the future, however, it is important that the sodium content of food products be lowered. In order for this to happen, the salt used during cooking needs to be limited in combination with increased availability and use of sodium-reduced products (20). If sodium levels in food products were to be lowered by 10–25%, it would most likely not be noticed by the consumer (21). Finland is an example of a country where salt intake has been successfully lowered. One of the actions taken to achieve this goal was cooperation with the food industry (22), a measure that would most likely be needed in Sweden also.

Vitamin D also showed low intake levels. Although the children received 31% of their daily intake of vitamin D from the school lunch, their intake still did not reach the recommended level. For school lunches to reach 30% of the new recommendation in the NNR, that is, 10 µg of vitamin D/day (7), it is likely that other measures will need to be taken than simply serving vitamin D-rich food items. Vitamin D-rich food sources are relatively few, and therefore enhanced food fortification could be an alternative (23). The iron content of school lunches also needs to be increased, considering the importance of this mineral for growing children and adolescents (7).

When it comes to sucrose, the *Riksmaten barn* food consumption survey showed that Swedish children in general eat too much added sugar during the day (3). This result was not found for school meals. Although the presented intakes of sucrose include both added sugars and natural sources of sucrose, the intake of sucrose was still below the NNR reference value for added sugars, and school meals contributed to 11% of the children's daily intake of sucrose. This indicates that the content of added sugars in Swedish school meals is low and that the main intake of sucrose occurs at other times during the day.

In general, the children in Grade 5 had a more difficult time reaching the reference values, especially regarding mineral intake. However, they generally showed a tendency to receive a proportionally higher energy and nutrient intake from school lunches during the day than children in Grade 2, indicating the importance of the school lunch for this group. In order to determine these differences between the two age groups, it would be necessary to take a closer look at school lunch menus and what food items served are actually consumed by the children. A Finnish study of adolescents showed that, although most pupils had the main course when having school lunch in the canteen, other components of the meal such as milk, salad, and bread were often omitted (24). The present study did not look at the types of foods consumed by the pupils and it is possible that the children did not reach some of the reference values because they omitted some components of the meal. For instance, the failure of the older age group to reach the reference value for calcium could be due to them choosing not to have milk with their school lunch, but this calls for further studies. In the present study, 32% of the children reported that they omit the main meal at least once per week, that is, they only have bread, salad, a beverage, and/or sour milk for lunch.

It is also possible that children failing to reach the reference values for several nutrients may be due to the children underestimating their intake. In the *Riksmaten barn* survey it was concluded that both age groups in general probably underestimated their daily food intake, and this tendency was especially evident among the fifth graders. It was estimated that 6% of the children in Grade 2 underestimated their energy intake as compared to 25% among the children in Grade 5 (3). Thus, the children in Grade 5 not reaching the reference values could also be due to underreporting. Moreover, the children recorded their intakes themselves or with the help of an adult. Depending on how much help the children received, the children recording their own intakes could also be a source of error.

This study is unique in that it looked at the energy and nutrient intakes of Swedish children from school meals by using a food record on a large and nationally representative sample, with a fairly high participation rate. However, the data collection took place in 2003, and it is possible that the situation looks different now, although recent data have shown similar results (13). The data were also collected before the law on nutritious school meals was enacted, and thus schools were not required at the time to follow the guidelines. It must also be stressed that the present study included more nutrients than what is required by the guidelines. However, it was of interest to study more nutrients than those stated in the guidelines, as the present study showed that the main problem nutrients of Swedish school meals are included in the guidelines. Thus, if the guidelines are fulfilled, we can expect the nutritional contribution from nutrients not included in the guidelines to be good as well. In the future, it would be of interest to perform a study in order to see whether the law requiring school meals to be nutritious has made a difference or not. The present study was based on secondary analyses on data from *Riksmaten barn* and the original aim of conducting *Riksmaten barn* was not primarily to study the children's intake from school meals. In future studies with this as the main goal, it would be appropriate to include more detailed data, for example school meal menus, and to design the study in a different way, that is, looking more at meal quality and what parts of the meal are omitted as well as at underreporting. This would also allow for future comparisons in follow-up studies.

According to the guidelines, an average of 4 weeks of school lunches should provide for the nutrients required, whereas in the present study a maximum of four school lunches were included, which may have affected the results. Moreover, since the children had eaten between one and four school lunches, the mean intake was used. As a result some information was lost. However, in order to make use of the full information from all meals for each individual, more advanced statistical methods for repeated measurements would be needed. For the purpose of this study, in order to compare the intake with the guidelines, using the mean intake was an adequate approach.

The food and beverages consumed at school have a great potential to affect the health of young people (25). The present study showed that children in Sweden receive close to or above 30% of their daily intake of nutrients from school meals, but that there is also room for improvement. The law prescribing that school meals must be nutritious has opened up new ways to ensure the nutritional quality of Swedish school meals. However, monitoring and evaluation are also important factors in ensuring quality (25). According to the Swedish Schools Inspectorate, the authority that supervises compliance with the law on nutritious school meals, this is one of the main problems with school meals and the municipalities need to take firmer action to make sure that the school meals served are nutritious (26). If this is done, the new law requiring school meals to be nutritious may enable the improvements needed for Swedish school meals to be made, so as to comply with the nutritional guidelines and thereby contribute to healthy dietary habits.
